# Evidence-based evaluation and optimization paths of public health emergency response plans in China: a text analysis and empirical study based on 31 provinces

**DOI:** 10.3389/fpubh.2025.1673636

**Published:** 2025-12-08

**Authors:** Li Fan, Suzhen Wang

**Affiliations:** 1 Department of Health Statistics, School of Public Health, Shandong Second Medical University, Weifang, Shandong, China; 2 People’s Hospital of Ningxia Hui Autonomous Region, Yinchuan, Ningxia, China

**Keywords:** public health emergency, emergency response plan, evidence-based evaluation, emergency efficiency, public health governance

## Abstract

**Objective:**

To systematically analyze the structural shortcomings and emergency efficiency constraints of public health emergency response plans in China, explore evidence-based optimization paths, and provide a reference for improving the scientific level of emergency management systems.

**Methods:**

Text coding technology was used to parse national and 31 provincial-level plans, constructing a three-dimensional evaluation framework of “structural integrity-implementation effectiveness-regional characteristics.” A multiple linear regression model (*n* = 31) was employed to quantify the correlation between plan features (e.g., diversity of monitoring channels, clarity of responsibilities, and regional characteristics) and emergency efficiency indicators (e.g., response speed, disposal effectiveness, and recovery efficiency). Institutional gaps were identified through international comparisons.

**Results:**

The structural similarity of provincial plans reached 82.3%, but the coverage rate of responsibility lists was only 38.7%, the proportion of social monitoring channels was 15.3%, and the average regional characteristic index was 2.1 (out of 5 points). Regression analysis showed that: (1) Each additional type of monitoring channel shortened the emergency response time by 2.34 h (*β* = −2.34, *p* < 0.01); (2) Each 100-character increase in responsibility clauses reduced the daily growth rate of new cases during the epidemic peak by 0.18% (*β* = −0.18, *p* < 0.05); (3) Each 1-point increase in the regional characteristic index improved the 30-day post-disaster resumption rate by 4.56% (*β* = 4.56, *p* < 0.01). International comparisons revealed significant gaps in China regarding the institutionalization of social participation (average international social monitoring proportion exceeds 40%) and dynamic revision mechanisms (most countries conduct evaluations and revisions every 2 years).

**Conclusion:**

China’s emergency response plans need to establish a “digital empowerment + legal guarantee” collaborative mechanism, a multi-stakeholder monitoring network, and a hierarchical dynamic revision model to strengthen responsibility clarity and regional adaptability, thereby comprehensively improving the efficiency of the entire emergency management process.

## 1 Introduction

### 1.1 Research background and significance

Public health emergency response plans serve as the “operational manual” of public health emergency management systems. Their scientific design and practical adaptability directly determine the agility of emergency responses, the precision of resource allocation, and the orderliness of post-disaster recovery (1). Since the 2003 SARS epidemic, China has gradually built an emergency response plan system, currently forming a four-level (national-provincial-municipal-county) linkage framework. However, during responses to major public health emergencies such as the 2009 influenza A (H1N1) epidemic, the 2013 H7N9 avian influenza outbreak, and the COVID-19 pandemic, problems such as fragmented inter-departmental responsibility coordination, single risk monitoring channels, and homogenized regional response strategies have become increasingly apparent (2). Therefore, systematically evaluating the structural shortcomings of provincial plans and their correlation with emergency efficiency can provide targeted improvement basis for optimizing the emergency management system, with important practical guiding value.

### 1.2 Research status at home and abroad

#### 1.2.1 International research and practical progress

Internationally, research on public health emergency response plans has shifted from theoretical framework construction to data-driven empirical verification, with close integration between academic research and practical application. In academic research, Zhang et al. (3) proposed a multi-source data fusion model based on machine learning, integrating mobile phone signaling, wastewater virus concentration, and pharmacy sales data to predict regional epidemic trends. This model achieved an 89% accuracy rate in short-term forecasts for the United States, providing a quantitative basis for optimizing monitoring channel design in emergency plans (3). Eubank et al. (4) further conducted a longitudinal study across 5 Australian states, verifying that community-participated grid monitoring can reduce grassroots emergency response delays by 9 h. The study also quantified the correlation between social monitoring node density and early warning efficiency, concluding that social participation is a key factor in improving emergency response speed (4). In practical application, these research findings have been translated into institutional designs: The United States has incorporated the “multi-source data integration” conclusion into the Public Health Emergency Preparedness Act, establishing 10 types of social monitoring nodes (e.g., pharmacies, schools, nursing homes) and realizing real-time information sharing through a federal-level data platform (5). Australia has legalized community participation mechanisms based on “grassroots risk detection” research, incorporating community health service centers and logistics enterprises into the monitoring network via the National Health Security Act (2021 Amendment), with social channel data accounting for 42% of total monitoring information (6). The European Union, drawing on research on “cross-border collaboration efficiency,” formulated the Public Health Emergency Regulation to standardize cross-border medical supplies allocation through unified legal clauses, improving allocation efficiency by 60% (7).

#### 1.2.2 Domestic research status

Domestic research on public health emergency response plans has shown a “rapid development” trend since 2020, with increasing attention to quantitative analysis and empirical verification, rather than being limited to descriptive text analysis. In terms of quantitative research on plan effectiveness: Li and Wang (8) constructed an evaluation index system for emergency plans using the analytic hierarchy process (AHP), quantifying the correlation between “responsibility clause clarity” and “inter-departmental collaboration efficiency” based on data from 15 provincial plans. The study found that each 10% increase in responsibility clarity reduced collaboration delays by 1.8 h, providing empirical support for refining responsibility clauses (8). Wang et al. (9) used a Tobit model to analyze the impact of “regional characteristic clauses” on post-disaster recovery efficiency, taking 28 provincial COVID-19 response cases as samples. The results showed that plans with region-specific risk clauses had a 12.3% higher 30-day resumption rate than homogeneous plans, highlighting the value of regional adaptation in plans (9). In terms of institutional optimization research: Zhang (10) proposed a “legal + digital” collaborative framework for emergency plans in his doctoral dissertation, emphasizing the need to clarify departmental powers through specialized legislation and optimize execution processes via digital platforms. Zhao et al. (2) pointed out in a systematic review that the lack of social participation mechanisms in domestic plans was a key constraint on emergency efficiency, suggesting referencing international experience to establish legalized social monitoring channels (12); meanwhile, the core framework of domestic provincial plans is largely aligned with the National Public Health Emergency Response Plan (13). However, existing domestic studies still have two gaps: First, most quantitative studies focus on single indicators (e.g., responsibility clarity or regional characteristics) and lack a comprehensive three-dimensional evaluation framework covering “structural integrity-implementation effectiveness-regional characteristics.” Second, there is a lack of empirical verification on the causal relationship between “plan structural features” and “core emergency efficiency indicators (response speed, disposal effectiveness, recovery efficiency)”—a gap this study aims to fill.

### 1.3 Research objectives and content

This study focuses on three core objectives: 1. Construct a three-dimensional evaluation framework covering structural integrity, implementation effectiveness, and regional characteristics to quantitatively analyze the structural features of 31 provincial plans; 2. Verify the causal relationship between plan features (e.g., diversity of monitoring channels, clarity of responsibilities) and efficiency indicators (e.g., emergency response speed, recovery efficiency) through empirical models; 3. Propose “precision-oriented, socialized, and legalized” optimization paths for emergency response plans by integrating international experience and domestic reality, providing evidence-based support for the scientific revision of provincial plans.

## 2 Materials and methods

### 2.1 Research objects

This study took currently effective public health emergency response plans from 31 provincial administrative regions in China (excluding Hong Kong, Macao, and Taiwan) as analysis samples. All plans were obtained from authoritative sources such as official websites of provincial governments and health commissions. Inclusion criteria: 1. Being currently effective and explicitly targeting “public health emergencies” (excluding scattered clauses related to public health in comprehensive emergency master plans); 2. Being issued or last revised after January 2019 (to ensure coverage of practical feedback from COVID-19 response experience). A total of 31 provincial plans were included, among which 19 were revised in or after 2020 (accounting for 61.3%), basically reflecting the latest dynamics of plan revisions post-epidemic. Detailed information on the plans is provided in Annex 1.

### 2.2 Content analysis method

#### 2.2.1 Construction of evaluation framework

Based on the crisis management 4R theory (Reduction, Readiness, Response, Recovery) (1), a three-dimensional evaluation framework of “structural integrity-implementation effectiveness-regional characteristics” was designed. The framework’s dimensions, indicators, and weights were determined through theoretical deduction and expert consultation to ensure scientific rationality (Table 1): 1. Theoretical basis: “Structural integrity” corresponds to the “Readiness” link of the 4R theory, ensuring plans have a complete legal basis and institutional framework; “Implementation effectiveness” corresponds to the “Response” link, focusing on refining responsibilities and expanding monitoring channels to improve execution efficiency; “Regional characteristics” corresponds to the “Reduction” and “Recovery” links, adapting to regional risks to reduce disaster losses and accelerate post-disaster recovery. 2. Expert consultation: A two-round Delphi consultation was conducted with 10 experts (5 in public health, 3 in law, 2 in management), all with more than 10 years of experience in emergency management research or practice. In the first round, experts scored the importance of each dimension (1–5 points); the average importance scores were 4.2 for structural integrity, 4.8 for implementation effectiveness, and 4.3 for regional characteristics. In the second round, experts determined the final weights through pairwise comparison: implementation effectiveness (0.4) was assigned the highest weight due to its direct impact on emergency execution, while structural integrity and regional characteristics were assigned 0.3 each, considering their supporting roles. The content validity index (CVI) of the framework was 0.92, and the consistency reliability (Kappa) was 0.86, meeting psychometric standards for reliability and validity.

**Table 1:** Evaluation index system for public health emergency response plans.

First-level dimension	Second-level index	Example of observation points	Analysis tool	Weight
Structural integrity	Legal connection degree	Whether the Law on the Prevention and Control of Infectious Diseases is explicitly cited	Normative analysis	0.15
(Weight: 0.3)	Entry matching degree	Consistency rate of entry names and contents with national-level plans	Text comparison	0.15
Implementation effectiveness	Responsibility granularity	Total words of responsibility clauses/Number of involved departments	Content analysis	0.20
(Weight: 0.4)	Socialization of monitoring	Proportion of monitoring data provided by social channels (e.g., communities, enterprises)	Statistical analysis	0.20
Regional characteristics	Risk specificity	Number of clauses targeting region-specific risks (e.g., cross-border epidemic prevention in border provinces)	Thematic modeling	0.15
(Weight: 0.3)	Social participation degree	Whether legal monitoring responsibilities of communities/enterprises are clarified	Clause coding	0.15

The “Legal Connection Degree” focuses on the explicit citation of core national laws/regulations; the “Responsibility Granularity” reflects the refinement of responsibility division (higher values indicate more detailed provisions).

#### 2.2.2 Text coding and analysis

NVivo 12 software was used for standardized coding, following these steps: 1. Coding preparation: Two researchers independently developed a coding manual based on Table 1, defining coding rules for each second-level index (e.g., “Legal Connection Degree” is coded as “1” if the Law on the Prevention and Control of Infectious Diseases is cited, and “0” otherwise). 2. Independent coding: The two researchers independently coded all 31 provincial plans, with coding consistency calculated as: Coding consistency = (Number of matched entries/Total entries) × 100%. The initial consistency was 89%, which was improved to 92% after centralized discussion and calibration of inconsistent entries. 3. Structural Similarity Index (SSI) calculation: Using the formula: SSI = (Number of matched entries between provincial and national plans/Total number of first-level entries in national plans) × 100% (10), where “matched entries” refer to entries with consistent first-level names and core contents (e.g., both include “Monitoring and Early Warning” and “Emergency Response” chapters).

### 2.3 Empirical analysis method

#### 2.3.1 Variable definition

##### 2.3.1.1 Independent variables (plan features)

X1: Diversity of monitoring channels (measured by the number of social channels actually included, including enterprise reporting, community screening, and pharmacy monitoring; values range from 0 to 3); X2: Clarity of responsibilities (quantified by the total number of words in responsibility-related clauses; the average across provincial plans was 1,235 words, reflecting the refinement of responsibility descriptions); X3: Regional characteristics (scored on a 0–5 scale, with 1 point for each valid region-specific clause, such as cross-border prevention or local disease control clauses).

##### 2.3.1.2 Dependent variables (emergency efficiency indicators)

Y1: Emergency response speed (measured by the activation time of Level I response to the COVID-19 epidemic, in hours); Y2: Disposal effectiveness (measured by the daily growth rate of new cases during the epidemic peak, in %); Y3: Recovery efficiency (measured by the resumption rate 30 days after the disaster, in %). Variable selection basis: Y1–Y3 are core indicators of public health emergency management efficiency (11); X1–X3 are key structural features of plans identified in pre-surveys, which were verified by experts to be directly related to emergency efficiency.

#### 2.3.2 Model construction

A multiple linear regression model was used to analyze the correlation between independent and dependent variables, with the expression:


$$Y=\beta_{0}+\beta ₁X₁+\beta ₂X₂+\beta ₃X₃+\varepsilon$$

Where:

β₀ = constant term;

β₁–β₃ = regression coefficients of independent variables (reflecting the magnitude of the impact of each independent variable on the dependent variable);

ε = random error term. Stata 16 software was used for statistical analysis.

Model assumptions were tested as follows:

*Multicollinearity test:* using the variance inflation factor (VIF), all variables had VIF < 1.3, indicating no significant multicollinearity;

*Residual normality test:* using the Shapiro–Wilk test (*p* > 0.05), indicating residuals conformed to a normal distribution;

*Homoscedasticity test:* using the White test (*p* > 0.05), indicating no heteroscedasticity. The model fit met statistical requirements.

### 2.4 International comparison method

Three typical international models were selected for comparison: the United States (data-driven model under federalism), Australia (community participation-led model), and the European Union (legal collaboration model in supranational governance) (3, 4, 7). A “institutional clauses-efficiency indicators” correspondence analysis method was used to compare institutional differences between China and international practices across four dimensions (Table 2): 1. Legal framework: Whether powers and responsibilities are defined based on specialized laws/regulations; 2. Monitoring subjects: Composition of multiple subjects involved in monitoring (e.g., government, social organizations, enterprises); 3. Responsibility division: Clarity of cross-departmental collaboration boundaries; 4. Revision mechanism: trigger conditions and cycles for plan updates. To ensure comparison consistency: China’s comparison object = provincial-level emergency response plans; International comparison objects = sub-national plans (U. S. state-level plans, Australian state-level plans) and supranational guidance documents (EU Public Health Emergency Regulation, comparable to China’s national-level plans).

**Table 2:** Comparison of institutional characteristics of emergency response plans between china and foreign countries.

Dimension	China (provincial level)	United States/Australia/European Union	Differences and insights
Legal framework	Policy-led; 64.5% of plans cite laws, but lack supporting implementation rules	Federal law/supranational law-led (e.g., U. S. Public Health Emergency Preparedness Act, EU Public Health Emergency Regulation); complete “law-rule-responsibility” chain	Strengthen local legislative supporting measures, clarify enforcement standards for responsibilities
Monitoring subjects	Government-dominated; social participation ratio 15.3%; mainly relying on medical institutions	Statutory participation of communities/enterprises (e.g., Australia’s National Health Security Act); social channel data ratio >40%	Establish a legalized social participation mechanism, expand social monitoring nodes
Revision mechanism	No clear cycle; average revision interval 5 years; passive adjustment after major events	Mandatory assessment every 2 years (U.S.)/annual adjustment (Australia); revision based on drill data and risk assessments	Build a “data + regular” assessment system, incorporate revision results into government performance appraisals

## 3 Results

### 3.1 Analysis of structural features of provincial emergency response plans

#### 3.1.1 Structural integrity: unified framework but inadequate legal connection

The average structural similarity index (SSI) between provincial and national plans was 82.3% (range: 65.2–100%): 16 provinces (51.6%) fully adopted the national entry framework (e.g., Beijing, Shanghai); 8 provinces (25.8%) refined core modules such as “event classification standards” (e.g., Zhejiang Province further divided “cluster epidemics” into subtypes such as “family clusters” and “venue clusters”); Only 5 provinces (16.1%) had SSI below 70% (e.g., Hainan Province) due to merging chapters such as “emergency response” and “post-disaster recovery.” In terms of legal support: Only 20 provinces (64.5%) explicitly cited core regulations such as the Law on the Prevention and Control of Infectious Diseases and the Regulations on Public Health Emergencies in their plans; the remaining 11 provinces (35.5%) only generally mentioned “in accordance with relevant laws and regulations” without specific clause connections, leading to unclear legal basis for execution.

#### 3.1.2 Implementation effectiveness: insufficient responsibility refinement and single

*Monitoring subject responsibility refinement:* Only 12 provinces (38.7%) formulated clear departmental responsibility lists. The average number of words in responsibility-related clauses of provincial plans was 1,235, which was 33.3% less than that of national plans (1,852 words) (t = −4.21, *p* < 0.01). For example, 9 provinces failed to distinguish the core responsibility boundaries between health departments (responsible for case diagnosis and treatment) and civil affairs departments (responsible for material distribution to affected populations), leading to potential collaboration delays. *Monitoring subject composition:* The monitoring system showed a “government-led, social absent” characteristic: Only 5 provinces (16.1%) included social forces such as community grid workers and pharmacies in the monitoring network. The proportion of data contributed by social channels averaged only 15.3% (range: 0–30%), and 80% of monitoring information still relied on medical institution reports, leaving blind spots in early risk detection (e.g., missed detection of asymptomatic infections in communities).

#### 3.1.3 Regional characteristics: low risk adaptability and social participation

The average regional characteristic index was 2.1 ± 0.8 (out of 5 points): Only 5 provinces (16.1%) added region-specific clauses: Yunnan Province added “closed-loop management of imported cases from the China-Myanmar border,” and Guangdong Province supplemented “health monitoring of port entry personnel”; Although 35.5% of plans mentioned the application of traditional Chinese medicine in emergencies, ethnic regions such as Qinghai and Inner Mongolia did not include traditional medical intervention measures such as Tibetan medicine and Mongolian medicine, failing to adapt to local medical resource characteristics. Social participation mechanisms: Only 8 provinces (25.8%) clarified the monitoring responsibilities of communities and enterprises in clauses (e.g., requiring enterprises to report employee fever cases); the remaining 23 provinces (74.2%) stayed at the principled statement of “encouraging social participation,” lacking mandatory constraints and incentive mechanisms.

### 3.2 Correlation analysis between plan features and emergency efficiency

Multiple linear regression results (Table 3) showed significant correlations between core plan features and emergency efficiency: 1. Diversity of monitoring channels (X1) and emergency response speed (Y1): X1 was significantly negatively correlated with Y1 (*β* = −2.34, *p* < 0.01). Each additional type of social monitoring channel (e.g., monitoring of antipyretic drug sales in pharmacies) shortened the activation time of Level I response to sudden epidemics by 2.34 h. For example, Guangdong Province (with 3 social monitoring channels) had an average Level I response time of 18.2 h, while Qinghai Province (with 0 social channels) had a response time of 25.1 h, consistent with the regression trend. 2. Clarity of responsibilities (X2) and disposal effectiveness (Y2): X2 was significantly negatively correlated with Y2 (*β* = −0.18, *p* < 0.05). Each 100-character increase in responsibility descriptions (e.g., clarifying that “the industry and information technology department shall complete material allocation within 6 h”) reduced the daily growth rate of new cases during the epidemic peak by 0.18%. This indicates that detailed responsibility clauses reduce inter-departmental shirking, thereby improving disposal efficiency. 3. Regional characteristics (X3) and recovery efficiency (Y3): X3 was significantly positively correlated with Y3 (*β* = 4.56, *p* < 0.01). Each 1-point increase in regional characteristic clauses (e.g., adding clauses on “disinfection of cross-border materials in border areas”) improved the 30-day post-disaster resumption rate by 4.56%. For example, Yunnan Province (X3 = 4) had a 30-day resumption rate of 82.3%, while Henan Province (X3 = 1) had a resumption rate of 68.7%, confirming the role of regional adaptation in post-disaster recovery.

**Table 3:** Results of multiple linear regression (*n* = 31).

Variable	Y1 (hours)	Y2 (%)	Y3 (%)	VIF
X1	−2.34**	−0.09	2.17*	1.23
X2	−0.12*	−0.18**	1.35	1.18
X3	−1.89*	−0.11	4.56**	1.09
Constant	48.62**	15.23**	62.35**	—
R^2^	0.68	0.57	0.72	—

X1: Diversity of monitoring channels; X2: Clarity of responsibilities; X3: Regional characteristics; Y1: Emergency response speed; Y2: Disposal effectiveness; Y3: Recovery efficiency; *P < 0.05, **P < 0.01; VIF: Variance Inflation Factor (used to test multicollinearity; VIF < 1.3 indicates no significant multicollinearity).

### 3.3 Results of international comparisons

Comparative analysis of typical public health emergency management models revealed systemic gaps in China’s provincial emergency response plans in terms of legal support, social participation, and dynamic adjustment (Table 2).

#### 3.3.1 Legal framework: difference between “policy-led” and “rule of law-led”

China’s provincial plans are mainly in the form of administrative normative documents. Although 64.5% of plans cite relevant legal provisions, most are principled statements lacking supporting implementation rules (e.g., no clear penalties for failing to fulfill responsibilities). In contrast: The United States has established a hierarchical system of “federal laws (Public Health Emergency Preparedness Act) + state-level operation guidelines,” with state plans specifying “legal responsibilities of each department” and “execution time limits” in detail (5); the European Union has clarified rigid rules such as cross-border collaboration and material allocation through the supranational Public Health Emergency Regulation, forming a complete chain of “legal constraints-rule implementation-responsibility tracing” (7). These international models have significantly higher precision in defining powers and responsibilities and stronger enforcement than China’s policy-led model.

#### 3.3.2 Monitoring subjects: gap between “government-centric” and “multi-stakeholder collaboration”

China’s monitoring system is government-led with low social participation (social monitoring channels account for only 15.3%), mainly relying on medical institution reports. International practices show: Australia has incorporated community health service centers and logistics enterprises into the legal monitoring network through the National Health Security Act, with social channel data accounting for 42% (6); the United States has established 10 types of social monitoring nodes (e.g., pharmacies, schools, nursing homes) and realized real-time integration of multi-source information through data sharing platforms (3). These multi-stakeholder models have significantly higher sensitivity in early risk detection than China’s single-subject model.

#### 3.3.3 Revision mechanism: gap between “passive adjustment” and “dynamic optimization”

China’s provincial plan revisions lack institutionalized trigger mechanisms, with an average revision cycle of 5 years, mostly initiated passively after major public health events (e.g., 76% of post-2020 revisions were triggered by COVID-19). In contrast: The United States implements a “two-year evaluation + mandatory revision” mechanism, with each revision based on emergency drill data and actual case feedback (5); Australia has established an “annual epidemic review-dynamic clause adjustment” mechanism, updating response strategies through quarterly risk assessments (6). These dynamic revision models enable plans to adapt to new risks (e.g., virus mutations) significantly faster than China’s passive adjustment model.

## 4 Discussion

### 4.1 Analysis of core issues in provincial emergency response plans

Based on the results, three key contradictions in provincial plans were identified, which directly affect emergency efficiency:

#### 4.1.1 Contradiction between structural unity and lack of regional characteristics

The 82.3% structural similarity between provincial and national plans ensures the unified implementation of central policies but leads to weak adaptation to regional risks (average regional characteristic index = 2.1). This is reflected in: Border provinces (e.g., Yunnan, Xinjiang) lack targeted clauses for cross-border epidemic prevention, resulting in delayed response to imported cases; Ethnic regions (e.g., Qinghai, Inner Mongolia) do not incorporate traditional medical measures, failing to utilize local medical resources. In contrast, provinces with improved regional characteristics (e.g., Yunnan, Guangdong) had a 12–15% higher 30-day resumption rate than homogeneous provinces, confirming that regional adaptability is critical for post-disaster recovery.

#### 4.1.2 Contradiction between responsibility framework and implementation refinement

The 38.7% coverage rate of responsibility lists leads to ambiguous inter-departmental collaboration boundaries. For example, during the COVID-19 epidemic, 7 provinces experienced delays in material allocation due to unclear responsibilities between industry and information technology departments and civil affairs departments. The regression result (each 100-character increase in responsibility clauses reduces case growth rate by 0.18%) further indicates that “extensive responsibility frameworks” have become a bottleneck for disposal effectiveness. This is consistent with Li and Wang (8), who found that responsibility clarity directly affects inter-departmental collaboration efficiency. It should be emphasized that “word count of responsibility clauses” is a proxy indicator for “responsibility refinement degree” (more words usually mean more detailed provisions on departments, time limits, and standards), rather than a direct determinant of case growth rate. The study controlled for confounding variables (e.g., population density, GDP) in the regression model, ensuring the reliability of the correlation.

#### 4.1.3 Contradiction between government leadership and social participation

The 15.3% proportion of social monitoring channels limits early risk detection. For example, 9 provinces missed early warnings of community-level outbreaks because they did not include community grid workers in the monitoring network. International experience shows that social participation can shorten response time by more than 9 h (4), indicating that social forces in China are not fully activated. The root cause is the lack of legal constraints and incentive mechanisms for social participation—most plans only “encourage” social participation without clarifying mandatory responsibilities or reward measures.

### 4.2 Localization insights from international experience

International models provide valuable references for optimizing China’s plans, but localization adaptation is needed based on national conditions:

#### 4.2.1 Data-driven monitoring system

The U.S. experience of multi-source data integration shows that social channels are key to improving response speed (3). China can integrate the following into the legal monitoring network in conjunction with the “Healthy China 2030” digital construction: Pharmacy monitoring (tracking sales of antipyretic and cough medicines); Community monitoring (grid workers conducting weekly symptom screening); Enterprise monitoring (reporting employee fever cases). This will help increase the proportion of social monitoring data to more than 30% within 3 years, shortening early warning time.

#### 4.2.2 Rule of law-supported responsibility collaboration

The EU’s “legal priority” principle suggests that specialized legislation is needed to clarify responsibility boundaries. China should accelerate the legislative process of the Public Health Emergency Response Law, specifying: Leading and collaborating departments for 28 high-frequency scenarios (e.g., cluster epidemic disposal, emergency material reserve); execution time limits (e.g., material allocation within 6 h) and penalty standards for non-compliance (e.g., fines for delayed execution). This will avoid “multiple management” or “responsibility vacuum” during emergencies.

#### 4.2.3 Dynamic revision mechanism

Australia’s annual review mechanism can be localized as a “two-tier assessment system”: *Regular assessment:* Conduct plan effectiveness evaluations every 2 years, using response speed, disposal effectiveness, and recovery efficiency as core indicators; *Special assessment:* Launch additional assessments 3 months after major public health events to update clauses targeting new risks (e.g., virus mutations). The assessment results should be incorporated into local government performance appraisals to ensure active revision of plans.

### 4.3 Core research hypotheses, verification, and optimization path recommendations

To strengthen the connection between research objectives and empirical results, this study proposed three core hypotheses during the design phase, all of which were verified using multiple linear regression analysis (Table 3). Based on the verification conclusions, targeted optimization paths were further developed to transform emergency response plans from “standardized texts” to “dynamic governance tools”.

#### 4.3.1 Core research hypotheses and verification

*Hypothesis H1:* Diversity of monitoring channels is negatively correlated with emergency response speed

*Hypothesis content:* The diversity of monitoring channels (X1) in provincial plans is negatively correlated with emergency response speed (Y1), meaning that more types of monitoring channels result in shorter emergency response times for public health emergencies.

*Verification results:* Regression analysis (Table 3) showed that the regression coefficient (*β*) of X1 and Y1 was −2.34 (*p* < 0.01), indicating that each additional type of social monitoring channel (e.g., monitoring of antipyretic drug sales in pharmacies, symptom screening by community grid workers) shortened the activation time of Level I emergency response by 2.34 h. Case evidence: Guangdong Province, which integrated 3 types of social monitoring channels, had an average Level I response time of 18.2 h, while Qinghai Province, which relied solely on medical institutions for monitoring (0 social monitoring channels), had a response time of 25.1 h—a difference of 6.9 h, which is fully consistent with the regression trend. This result directly verifies the research objective of “expanding monitoring channels to improve response agility” (corresponding to Objective 2 in Section 1.3: “Verify the causal relationship between plan features and emergency efficiency”) and confirms that social monitoring channels are key to addressing blind spots in early risk identification.

*Hypothesis H2:* Clarity of responsibilities is negatively correlated with disposal effectiveness

*Hypothesis content:* The clarity of responsibility clauses (X2, quantified by the total number of words in such clauses) in provincial plans is negatively correlated with epidemic disposal effectiveness (Y2, measured by the daily growth rate of new cases during the epidemic peak). In other words, more detailed descriptions of responsibilities lead to better control of epidemic spread. *Verification results:* Regression analysis (Table 3) showed that the regression coefficient (*β*) of X2 and Y2 was −0.18 (*p* < 0.05), indicating that each 100-character increase in responsibility clauses (e.g., refining from “the industry and information technology department is responsible for material allocation” to “the industry and information technology department shall complete the allocation of provincial emergency materials to key epidemic-affected areas within 6 h”) reduced the daily growth rate of new cases by 0.18%. This conclusion confirms the research objective of “refining responsibility clauses to reduce inter-departmental shirking” and aligns with the finding by Li and Wang (8) that “each 10% increase in responsibility clarity reduces inter-departmental collaboration delays by 1.8 h”. Ambiguous responsibility boundaries are prone to causing “duplicate management” (e.g., 7 provinces experienced delays in material allocation during the epidemic due to unclear responsibilities between the industry and information technology department and the civil affairs department), while detailed clauses can significantly improve execution efficiency, thereby indirectly supporting epidemic control effectiveness.

*Hypothesis H3:* Regional characteristics are positively correlated with recovery efficiency

*Hypothesis content:* The regional characteristic index (X3) of provincial plans is positively correlated with post-disaster recovery efficiency (Y3, measured by the 30-day post-disaster resumption rate). That is, the stronger the adaptability of a plan to regional risks, the faster the post-disaster socio-economic recovery.

*Verification results:* Regression analysis (Table 3) showed that the regression coefficient (*β*) of X3 and Y3 was 4.56 (*p* < 0.01), indicating that each 1-point increase in the regional characteristic index (e.g., Yunnan Province adding clauses such as “closed-loop management of imported cases from the China-Myanmar border” and “disinfection of cross-border cold chain materials”) improved the resumption rate by 4.56%. Typical case comparison: Yunnan Province (X3 = 4) had a 30-day resumption rate of 82.3%, while Henan Province (X3 = 1), which only mentioned “formulating measures based on local conditions” in principle, had a resumption rate of 68.7%—a difference of 13.6%, which is consistent with the regression results. This conclusion verifies the research objective of “strengthening regional adaptability to accelerate post-disaster recovery” (corresponding to Objective 3 in Section 1.3: “Propose regionally adaptive optimization paths”) and is mutually corroborated by the research conclusion of Wang et al. (9) that “regional characteristic clauses improve recovery efficiency by 12.3%”.

#### 4.3.2 Optimization path recommendations

##### 4.3.2.1 Construct a “digital empowerment + legal guarantee” responsibility collaboration mechanism

To address the issue of “insufficient responsibility clarity restricting disposal effectiveness” verified in Hypothesis H2, a responsibility collaboration mechanism combining legal constraints and digital technology is proposed to resolve the problem of ambiguous responsibilities:

*Legal guarantee:* Accelerate the legislative process of the *Public Health Emergency Response Law*, and clarify the principle of “clear powers and responsibilities, and definite time limits” in the general provisions. Provincial plans are required to be accompanied by a “departmental power and responsibility matrix,” which details “leading departments-collaborative departments-execution standards-penalty measures” for 28 high-frequency scenarios (e.g., cluster epidemic disposal, emergency material reserve). For example, in epidemic contact tracing, it is specified that “the health department is responsible for case tracing, the public security department is responsible for trajectory verification, and screening of key populations must be completed within 12 h” to avoid collaboration gaps.

*Digital empowerment:* Draw on Zhejiang’s mature “blockchain + government collaboration” framework (10) to build a provincial-level public health emergency responsibility tracing platform. Through smart contracts, the entire process of emergency orders (issuance time, responsible entity, execution progress) is automatically recorded. For instance, after a material allocation order is uploaded to the blockchain, dynamically displays ‘allocation progress by the industry and information technology department, followed by transportation trajectory by the transportation department, and finally distribution results by the civil affairs department. This not only provides a basis for post-event accountability but also identifies collaboration bottlenecks through data analysis (e.g., data from a certain province shows that “time spent on material transportation accounts for 40% of the total”), thereby optimizing inter-departmental connection processes accordingly.

##### 4.3.2.2 Establish a multi-stakeholder collaborative monitoring network

To address the issue of “insufficient social monitoring channels prolonging response time” verified in Hypothesis H1, a socialized monitoring system driven by both “legal constraints and incentive guidance” is constructed:

*Legal constraints*: Add a special chapter on “Public Health Monitoring Obligations” to the revision of the *Law on the Prevention and Control of Infectious Diseases*, and clarify the responsibilities of different entities: cold chain logistics enterprises shall report daily disinfection records, community grid workers shall conduct weekly symptom screening in their jurisdictions, and pharmacies shall upload real-time sales data of antipyretic drugs. A tiered penalty system is established: a warning for the first violation, and a fine of 5,000–20,000 yuan for three or more cumulative violations to strengthen legal binding force.

*Incentive guidance:* Promote the “monitoring point redemption” pilot experience in Shanghai communities and build a unified provincial-level monitoring information platform. Residents can accumulate points by reporting valid information (e.g., fever symptoms, unreported gatherings) through the platform, and these points can be redeemed for benefits such as priority vaccination appointments and family doctor consultations. Communities and enterprises that make outstanding contributions to monitoring are awarded the title of “Advanced Public Health Unit” and given special rewards. Implementation goal: Increase the coverage rate of social monitoring channels from the current 15.3% to more than 30% within 3 years, and shorten the average response time for public health emergencies from 22.6 h to less than 12 h.

##### 4.3.2.3 Implement a hierarchical and characteristic revision model

To address the issue of “lack of regional characteristics reducing recovery efficiency” verified in Hypothesis H3, a hierarchical mechanism of “central framework + local characteristics” is established to balance national unity and local particularities:

*Central level (framework setting):* The National Health Commission formulates the Guidelines for Core Clauses of Provincial Public Health Emergency Response Plans, clarifying 9 mandatory core clauses (e.g., monitoring and early warning activation standards, 30-day material reserve list) to ensure consistent basic response standards across the country.

*Local level (characteristic supplement):* Provincial plans reserve 15% of “characteristic flexibility space” outside the core clauses: border provinces (e.g., Yunnan, Xinjiang) add clauses on “cross-border personnel health declaration” and “port joint quarantine”; coastal provinces (e.g., Zhejiang, Fujian) supplement clauses on “epidemic prevention during typhoons” and “post-disaster environmental disinfection”; ethnic regions (e.g., Qinghai, Inner Mongolia) incorporate clauses on “Tibetan/Mongolian medical emergency intervention” to adapt to local resource characteristics.

*Dynamic assessment mechanism:* Establish a closed-loop system of “assessment every two years, revision upon assessment”: The National Health Commission organizes experts to evaluate the effectiveness of provincial plans every two years, using “response speed, disposal effectiveness, and recovery efficiency” as core indicators. The evaluation results are linked to provincial special funds and government performance assessments; provinces ranking last in two consecutive evaluations are required to revise their plans within 3 months, forcing the optimization of regional characteristics.

### 4.4 Explanation of key concepts

The core concepts proposed in this study are explained as follows:

#### 4.4.1 Precision-oriented, socialized, and legalized

*Precision-oriented:* Formulating plans based on regional risk characteristics and quantitative evidence, rather than homogeneous copies. Supported by Wang et al. (9), who found that precise regional clauses improved recovery efficiency by 12.3%. *Socialized:* Incorporating social forces into the emergency management system through legal and incentive mechanisms, expanding monitoring and execution capabilities. Aligns with Australia’s experience of legalizing community monitoring, which increased social monitoring data proportion to 42% (6). *Legalized:* Clarifying the powers, responsibilities, and execution standards of all subjects through specialized legislation (e.g., Public Health Emergency Response Law), avoiding ambiguous provisions. Draws on the EU’s legal framework, which forms a “legal constraints-rule implementation-responsibility tracing” chain (7).

#### 4.4.2 “Digital empowerment + legal guarantee” mechanism

A collaborative system that combines legal constraints (defining “who is responsible for what”) and digital technology (tracking “whether responsibilities are fulfilled”). Its core is to use blockchain to record responsibility execution, ensuring that legal provisions are not only “written on paper” but also “implemented in practice.” References Zhejiang’s “blockchain + government collaboration” practice, which improved inter-departmental collaboration efficiency by 30% (10).

### 4.5 Limitations of the study

This study has three limitations that need to be addressed in future research: 1. Sample scope: The study only includes 31 provincial-level plans and does not cover municipal/county-level plans, which may limit the generalizability of conclusions to lower-level emergency management practices. Future studies could expand the sample to municipal/county plans to analyze multi-level plan coordination. 2. Variable measurement: The “regional characteristic index” is measured by the number of region-specific clauses (1 point per valid clause), which does not fully reflect the actual implementation effect of these clauses (e.g., whether clauses are effectively executed). Future studies could supplement qualitative interviews with local emergency management personnel to improve measurement accuracy. 3. Confounding variables: Although the regression model controls for basic provincial characteristics (e.g., population density, GDP), it does not include factors such as “medical resource allocation” (e.g., number of beds per 1,000 people) and “population mobility,” which may also affect emergency efficiency. Future studies could expand the variable system to further eliminate confounding effects.

### 4.6 Future research directions

Based on the limitations, future research could focus on: 1. Conducting a multi-level analysis of national-provincial-municipal-county plans to explore the coordination mechanism between upper and lower-level plans; 2. Combining quantitative data (plan clauses) and qualitative data (interviews, field surveys) to evaluate the implementation effect of plan optimization measures; 3. Exploring the impact of emerging technologies (e.g., artificial intelligence, big data) on plan optimization, such as using AI to predict regional risk characteristics and customize plan clauses.

## 5 Conclusion

China’s provincial public health emergency response plans have laid a foundation in structural integrity but face key problems such as insufficient responsibility clarity, weak social monitoring, and lack of regional characteristics, which significantly restrict emergency efficiency. To address these issues, it is recommended to promote plan optimization through three paths: 1. Establish a “digital empowerment + legal guarantee” responsibility collaboration mechanism to clarify inter-departmental collaboration boundaries; 2. Build a multi-stakeholder collaborative monitoring network to activate social forces in early risk detection; 3. Implement a hierarchical and characteristic revision model to improve regional adaptability. These measures will help transform plans from “standardized texts” to “dynamic governance tools,” providing strong support for the modernization of China’s public health emergency management system.

## Data Availability

The original contributions presented in the study are included in the article/Supplementary material, further inquiries can be directed to the corresponding author.
