# Supplementary material for: Evidence-based evaluation and optimization paths of public health emergency response plans in China: a text analysis and empirical study based on 31 provinces
Source: Front Public Health. 2025 Dec 8;13:1673636. doi: 10.3389/fpubh.2025.1673636 (PMC12719516; doi:10.3389/fpubh.2025.1673636)
Supplement: Supplementary file 1 [file Data_Sheet_1.pdf]

Annex 1 List of Public Health Emergency Response Plans of 31 Provincial Administrative Regions in China

Province	Title of the Plan	Issuance/Last Revision Date	Source (Official Website)
Beijing	Public Health Emergency Response Plan of Beijing Municipality	Revised in September 2021	Beijing Municipal Health Commission
Tianjin	Tianjin Municipal Public Health Emergency Response Plan (2022 Version)	Revised in April 2022	Tianjin Municipal Government
Hebei	Hebei Provincial Public Health Emergency Response Plan	Revised in June 2020	Hebei Provincial Health Commission
Shanxi	Shanxi Provincial Public Health Emergency Response Plan (2021 Revision)	Revised in August 2021	Shanxi Provincial Health Commission
Inner Mongolia	Inner Mongolia Autonomous Region Public Health Emergency Response Plan	Revised in July 2020	Inner Mongolia Autonomous Region Health Commission
Liaoning	Liaoning Provincial Public Health Emergency Response Plan	Revised in March 2021	Liaoning Provincial Health Commission
Jilin	Jilin Provincial Public Health Emergency Response Plan (2022 Edition)	Revised in May 2022	Jilin Provincial Government
Heilongjiang	Heilongjiang Provincial Public Health Emergency Response Plan	Revised in September 2020	Heilongjiang Provincial Health Commission
Shanghai	Shanghai Public Health Emergency Response Plan (2022 Version)	Revised in March 2022	Shanghai Municipal Government
Jiangsu	Jiangsu Provincial Public Health Emergency Response Plan	Revised in November 2021	Jiangsu Provincial Health Commission
Zhejiang	Zhejiang Provincial Public Health Emergency Response Plan (2021 Update)	Revised in December 2021	Zhejiang Provincial Health Commission
Anhui	Anhui Provincial Public Health Emergency Response Plan	Revised in July 2020	Anhui Provincial Health Commission
Fujian	Fujian Provincial Public Health Emergency Response Plan (2022 Revision)	Revised in June 2022	Fujian Provincial Health Commission
Jiangxi	Jiangxi Provincial Public	Revised in August	Jiangxi Provincial

Province	Title of the Plan	Issuance/Last Revision Date	Source (Official Website)
	Health Emergency Response Plan	2020	Health Commission
Shandong	Shandong Provincial Public Health Emergency Response Plan	Revised in October 2021	Shandong Provincial Health Commission
Henan	Henan Provincial Public Health Emergency Response Plan (2022 Edition)	Revised in April 2022	Henan Provincial Government
Hubei	Hubei Provincial Public Health Emergency Response Plan	Revised in September 2020	Hubei Provincial Health Commission
Hunan	Hunan Provincial Public Health Emergency Response Plan (2021 Revision)	Revised in July 2021	Hunan Provincial Health Commission
Guangdong	Guangdong Provincial Public Health Emergency Response Plan	Revised in June 2020	Guangdong Provincial Health Commission
Guangxi	Guangxi Zhuang Autonomous Region Public Health Emergency Response Plan	Revised in August 2020	Guangxi Zhuang Autonomous Region Health Commission
Hainan	Hainan Provincial Public Health Emergency Response Plan (2022 Version)	Revised in May 2022	Hainan Provincial Government
Chongqing	Chongqing Municipal Public Health Emergency Response Plan	Revised in November 2021	Chongqing Municipal Health Commission
Sichuan	Sichuan Provincial Public Health Emergency Response Plan	Revised in July 2020	Sichuan Provincial Health Commission
Guizhou	Guizhou Provincial Public Health Emergency Response Plan (2021 Revision)	Revised in September 2021	Guizhou Provincial Health Commission
Yunnan	Yunnan Provincial Public Health Emergency Response Plan	Revised in June 2020	Yunnan Provincial Health Commission
Tibet	Tibet Autonomous Region Public Health Emergency Response Plan	Revised in August 2020	Tibet Autonomous Region Health Commission
Shaanxi	Shaanxi Provincial Public Health Emergency Response Plan (2022 Edition)	Revised in March 2022	Shaanxi Provincial Government

Province	Title of the Plan	Issuance/Last Revision Date	Source (Official Website)
Gansu	Gansu Provincial Public Health Emergency Response Plan	Revised in July 2020	Gansu Provincial Health Commission
Qinghai	Qinghai Provincial Public Health Emergency Response Plan (2021 Revision)	Revised in October 2021	Qinghai Provincial Health Commission
Ningxia	Ningxia Hui Autonomous Region Public Health Emergency Response Plan	Revised in September 2020	Ningxia Hui Autonomous Region Health Commission
Xinjiang	Xinjiang Uygur Autonomous Region Public Health Emergency Response Plan	Revised in August 2020	Xinjiang Uygur Autonomous Region Health Commission
